# National Learning Objectives in Dermatology and Venereology for the New Swedish Medical Programme

**DOI:** 10.2340/actadv.v106.44059

**Published:** 2026-01-23

**Authors:** Sam POLESIE, John PAOLI, Kari NIELSEN, Andreas SONESSON, Alexander SHAYESTEH, Anna JOSEFSON, Charlotta ENERBÄCK, Emma K. JOHANSSON, Torborg HOPPE

**Affiliations:** 1 Department of Dermatology and Venereology, Institute of Clinical Sciences, Sahlgrenska Academy, University of Gothenburg, Gothenburg, Sweden; 2 Region Västra Götaland, Sahlgrenska University Hospital, Department of Dermatology and Venereology, Gothenburg, Sweden; 3 Center for Digital Health, Sahlgrenska University Hospital, Gothenburg, Region Västra Götaland, Sweden; 4 Lund University Cancer Center, LUCC and Lund University Skin Cancer Research Group, LUSCaR Lund, Clinical Sciences Lund, Lund University, Sweden; 5 Division of Dermatology and Venereology, Department of Clinical Sciences Lund, Faculty of Medicine, Lund University, Lund, Sweden; 6 Department of Dermatology and Venereology, Skåne University Hospital, Lund, Sweden; 7 Department of Public Health and Clinical Medicine, Dermatology and Venereology, Umeå University, Umeå, Sweden; 8 School of Medical Sciences, Faculty of Medicine and Health, Örebro University, Örebro, Sweden; 9 Department of Dermatology, Örebro University Hospital, Örebro, Sweden; 10 Department of Biomedical and Clinical Sciences, Faculty of Health Sciences, Linköping University, Linköping, Sweden; 11 Division of Dermatology and Venereology, Department of Medicine Solna, Karolinska Institutet, Stockholm, Sweden; Department of Dermatology and Venereology, Karolinska University Hospital, Stockholm, Sweden; 12 Department of Medical Sciences, Dermatology and Venereology, Uppsala University, Uppsala, Sweden. Division of Dermatology and Venereology, Uppsala University Hospital, Sweden

**Keywords:** clinical competence, curriculum, dermatology/education, education, medical, undergraduate, learning, professional competence, sexually transmitted diseases/education, venereology

## Abstract

Effective medical education relies on clearly defined learning objectives that foster deep and meaningful learning. This article presents a consensus-driven proposal for national learning objectives in dermatology and venereology within Sweden’s new medical programme qualifying for licensure. The primary aim is to harmonize educational standards across all medical faculties and to establish a common understanding of the expected level of knowledge and competence at the point of medical licensure. Using a structured approach, existing curricula were analysed and categorized according to a modified version of Bloom’s taxonomy. The initial list of objectives and core conditions was then expanded through input from invited educators at all participating medical faculties as well as practising GPs. One designated assessor from each institution independently reviewed the expanded material to determine the expected knowledge level for each condition and to assess whether any items fell outside the intended scope. This process resulted in the identification of 36 learning objectives, along with a list of 124 core diagnoses and/or medications. The learning objectives encompass knowledge, practical skills, and professional attitudes in the management of dermatological and sexually transmitted diseases. The next step is to implement them with the aim of evaluating their impact on dermatology and venereology education. In this future work, student involvement should be prioritized to ensure a learner-centred approach throughout the process.

## SIGNIFICANCE

Effective teaching and meaningful learning stem from clear, student-centred objectives that promote engagement and deep understanding. Nationally standardized goals enhance consistency in medical education, support collaboration, and ensure comparable competencies among undergraduates in dermatology and venereology. This consensus-based proposal, developed by representatives from all Swedish medical faculties, outlines national learning objectives for the new medical programme qualifying for licensure. The aim is to establish a unified educational standard that guides teachers on what to teach students during their clinical training, and to support teachers’ continued professional development, thereby strengthening the quality and coherence of medical education in Sweden.

Sweden’s medical programme is currently undergoing major transformation. The new programme, designed to align more closely with the European educational model, extends from 11 to 12 semesters and leads directly to medical licensure (1). Consequently, medical licensure is now granted after 6 years of study, replacing the previous system of 5.5 years of education followed by at least 1.5 years of internship. This shift has effectively redefined the educational mission of Swedish medical universities. In the new curriculum, the dermatology and venereology course spans approximately 2–3 weeks at the different institutions (**Table I**).

**Table I:** Changes in dermatology and venereology education in the Swedish medical curriculum and teaching methods

University	Placement and scope of the course in dermatology and venereology in the former medical programme	Placement and scope of the course in dermatology and venereology in the new medical programme	Reduction in the scope of the course	Teaching methods/learning activities
Gothenburg	20 days during semester 9	11 days^a^ during semesters 5 and 9	45%	Recorded lectures, case-based seminars, online quizzes, practical workshops, clinical placement
Linköping	14 days during semester 10	14 days during semester 10	0%	Practice in the clinic, including outpatient clinics with a dermatologist, student outpatient clinics, case-based seminars, online training using Cyberderm and focused training in dermatoscopy
Lund	9 days^b^ during semester 7	9 days^b^ during semester 7	0%	Workplace-integrated learning, practical training on patients, and case-based teaching. Seminars, quiz, and workshops/skills training on skin surgery, treatment, and dermatoscopy
Karolinska (Stockholm)	1 day in semester 5 + 14 days in semester 6	15 days during semester 6	0%	Lectures (both live and recorded), Team-Based-Learning seminars (15), case-based seminars, workshops for practical training, workplace-integrated learning (practical training on patients in skin assessment and retrieving adequate medical history)
Umeå	15 days during semester 8	15 days during semester 8	0%	Theoretical learning with mandatory practical training over a 3-week period. Supervised clinical work and auscultations both in dermatology and venereology, seminars, case studies, workshops, telemedicine and quiz. Lectures are mostly prerecorded but also integrated in clinical work
Uppsala	13 days in dermatology + 1 day in venereology during semester 7	12.5 days in dermatology and venereology during semester 6	12%	Lectures, both live and recorded, case-based seminars, integrated theme days with rheumatology and pathology, quizzes, and workshops as well as practice-based training with student clinics, auscultations, and clinical seminars
Örebro	Semester 2: 6 teaching hours on “normal skin” Semester 4: 12 teaching hours on pathophysiology Semester 7: 19 teaching hours on clinical practice and treatment + ≈5 days of clinical placement (workplace-based learning)	Semesters 2 and 4: same structure Semester 7: short instructional videos + 15 teaching hours + 5 days of clinical placement (based on a 28-h week)	0%	Lectures, both live and recorded, case-based seminars, workshops for practical training, workplace-integrated learning, clinical placement

a One day of venereology during semester 5 and 10 days of dermatology during semester 9.

b Including workplace-integrated learning and case-based teaching.

Ireland et al. recently reported that medical students and junior doctors in Australia often express low confidence in managing dermatological conditions, largely due to insufficient clinical exposure and a lack of formal assessment. They advocate for the implementation of a standardized curriculum and expanded clinical teaching – recommendations that may also be pertinent to the Swedish context (2).

Programme directors across Sweden consistently emphasize that teaching should prioritize common conditions, serious ones, and those that require urgent care. We view this as a timely opportunity for cross-institutional collaboration. By agreeing on a national core curriculum in dermatology and venereology, we can establish a shared foundation for quality and equity within the educational system. Consequently, this is a high-priority issue that is best addressed through national consensus.

Within the Swedish Society of Dermatology and Venereology (SSDV), a dedicated interest group for research and undergraduate education has long discussed the value of establishing a national consensus on learning objectives that complements the more general learning outcomes defined by the national qualification framework. Furthermore, we have considered the benefit of compiling a detailed list of diagnoses, concepts, and therapies that constitute the core competencies in dermatology and venereology for a licensed physician.

## MATERIALS AND METHODS

In December 2023, work began on compiling the national learning objectives for dermatology and venereology. All existing documents relating to learning objectives and, where applicable, study guides from all 7 universities offering medical education leading to a licensure were collected. Based on these documents, a unified list of learning objectives was assembled.

The structure of the objectives followed the categories outlined in the Swedish Higher Education Ordinance’s qualification descriptors: *knowledge and understanding*, *competence and skills*, and *judgement and approach*.

The first draft of the learning objectives was compiled by the first author and then circulated to all co-authors for feedback. After several rounds of discussion, we ultimately reached a consensus on a final document comprising 36 learning objectives. No formal Delphi or majority-decision process was applied; instead, the learning objectives were formed purely by consensus. We defined “consensus“ as agreement among the authors, reached when no further substantive comments or objections were raised by any author. In other words, we considered consensus achieved when all authors were satisfied with the final decisions and no one had any remaining concerns. The authors from each university were encouraged to invite teachers from their respective medical faculty to contribute feedback on the learning objectives. Overall, 7 additional dermatologists involved in medical education provided feedback on the learning objectives.

In addition to the objectives, a comprehensive list was compiled of all diagnoses, concepts, and therapies mentioned across institutions. This updated list was then reviewed by one designated assessor from each institution.

At this stage, the assessors independently evaluated the appropriate level of knowledge expected of students for each condition, or whether a condition was considered beyond the expected scope and should be excluded. To classify the expected knowledge levels, a modified version of Bloom’s taxonomy was applied (3), distinguishing between: basic knowledge, composite/multifaceted knowledge, related knowledge, and transferable/extended knowledge (**Table II**). All faculties responded independently, and the final version was then compiled and redistributed for a final round of feedback.

**Table II:** Selectable elements concerning the grading of detailed learning objectives

*S0. Remove from the learning objectives*
*S1. Basic Knowledge* At this level, students are expected, for example, to be able to mention or recognize a term or concept, describe its meaning, list relevant factors, or delineate a phenomenon clearly enough to prevent misunderstandings – such as by defining a disease condition. This level is characterized by verbs such as *recognize*, *demonstrate knowledge of*, *define*, and *identify*
*S2. Multiple/Integrated Knowledge* At this level, students are expected, for example, to describe relationships, and to use terms, causes, or factors within a subject area in a coherent manner. This level is described using verbs such as *account for* or *describe*
*S3. Relational Knowledge* Students are expected to integrate facts into a meaningful context, to clarify relationships by linking causes and effects, and to compare phenomena by highlighting essential similarities and differences. This level is described using verbs such as *explain* and *compare*
*S4. Transferable/Advanced Knowledge* Students are expected to place core facts within broader and deeper contexts, to generalize and apply principles to new situations, and to discuss new phenomena based on previously acquired knowledge. This level is described using verbs such as *discuss*, *analyse*, *critically evaluate*, and *assess*

After the group had agreed on a final document, the learning objectives – along with the comprehensive list of diagnoses and the desired level of knowledge – were distributed to 9 physicians working in primary healthcare, to gather further feedback from the most important collaborative specialty for dermatologists. All authors were asked to consult colleagues in the primary healthcare setting, preferably those with academic affiliations. Both junior (*n* = 5) and senior physicians (*n* = 4) were invited to participate. We did not account for any specific geographic or urban/rural distribution among the GPs.

After reviewing the compiled feedback from colleagues in primary healthcare another round of discussions between the authors was initiated, and a final version was presented in November 2025.

## RESULTS

The process resulted in a total of 36 learning objectives, categorized as follows: knowledge and understanding (19 objectives), competence and skills (12 objectives), and judgement and approach (5 objectives) (**Table III** and Appendix S2 [*Swedish version*]). These learning objectives define the specific knowledge and competencies that physicians should attain in areas such as aetiology, diagnosis, treatment, and communication related to dermatological and venereological conditions, as well as professional attitudes and clinical judgement in the care of affected patients.

**Table III:** Learning objectives for dermatology and venereology in the new medical programme (English version)

*Knowledge and understanding*
**K1.** Knowledge of the aetiology and pathogenesis of various dermatological and venereological diseases (Appendix S1), including fundamental principles of skin biology (i.e., structure and function) such as skin homeostasis, the skin barrier function, cutaneous immunology, and vitamin D synthesis **K2.** Ability to clinically recognize and differentiate between inflammatory skin diseases, skin tumours, and sexually transmitted infections, including their key characteristics, to establish an accurate diagnosis (see Appendix S1) **K3.** Understanding of the pharmacological properties and side effect profiles of the most commonly used medications in the treatment of skin and venereological diseases (see Appendix S1) **K4.** Knowledge of diagnostic methods and their application in the assessment of dermatological and venereological conditions **K5.** Understanding of how genetic and environmental factors influence the development and progression of skin and venereological diseases **K6.** Basic knowledge of the epidemiology and prevalence of dermatological and venereological conditions both nationally and globally **K7.** Understanding of evidence-based guidelines and their role in the evaluation and treatment of skin and venereological diseases **K8.** Understanding of how dermatological and venereological diseases manifest differently in patients of varying skin types, ages, and sexes, and how these differences influence diagnosis and treatment **K9.** Knowledge of how cultural and social factors influence patients’ experiences of skin and venereological diseases **K10.** Understanding of multidisciplinary approaches and collaboration across medical specialties in managing complex dermatological and venereological conditions **K11.** Knowledge of urgent dermatological conditions (see Appendix S1) **K12.** Knowledge of preventive strategies and patient education to reduce the risks and consequences of skin and venereological disease **K13.** Understanding of how skin and venereological diseases impact patients’ health-related quality of life **K14.** Knowledge of ethical and legal considerations in dermatology and venereology, including communicable disease legislation, patient confidentiality, and informed consent **K15.** Understanding of the referral process, including essential content and formatting for effective communication between primary and specialist care **K16.** Knowledge of appropriate terminology in dermatology and venereology to ensure clear, consistent communication with colleagues and patients, and to support effective documentation and information exchange **K17.** Understanding of the principles of phototherapy (narrowband UVB, UVA) and light-based treatments (laser, IPL) used in dermatology and venereology **K18.** Understanding of the process and practice of contact tracing **K19.** Knowledge of therapeutic procedures, including surgical interventions and cryotherapy, for various skin and venereological diseases
*Competence and skills*
**F1.** Ability to perform and document a dermatological examination to identify various skin lesions, including inflammatory skin conditions, skin tumours, and sexually transmitted infections **F2.** Ability to perform a skin punch biopsy **F3.** Ability to use various diagnostic tools and methods, such as dermatoscopy and the ankle–brachial index **F4.** Ability to formulate relevant differential diagnoses based on clinical observations and laboratory findings **F5.** Ability to manage and follow up patients with chronic dermatological and venereological conditions to monitor disease progression and treatment effectiveness **F6.** Ability to communicate clearly and empathetically with patients about their dermatological or venereological conditions, treatment options, and prognosis **F7.** Ability to assess and manage skin and venereological diseases in patients with diverse skin types **F8.** Ability to manage and treat urgent dermatological conditions (see Appendix S1) **F9.** Ability to educate and advise patients on preventive measures to reduce the risk of skin and sexually transmitted diseases **F10.** Ability to assess and manage infectious dermatological and venereological conditions to prevent transmission and reduce risk to both individual patients and the broader community **F11.** Ability to tailor treatment strategies and therapies to individual patient needs and underlying factors **F12.** Ability to effectively assess and document dermatological and venereological diseases in order to write well-founded and accurate referrals to specialist care
*Judgement and approach*
**V1.** Identify and reflect on the importance of patient-centred care in improving treatment outcomes in dermatology and venereology **V2.** Demonstrate a reflective and professional attitude toward the patient’s cultural and social background in the diagnosis and treatment of dermatological conditions **V3.** Display a professional approach to ethical and privacy issues that may arise in the treatment and handling of patient information within dermatological and venereological care **V4.** Identify and reflect on how socioeconomic factors may influence access to care and treatment in dermatology and venereology **V5.** Demonstrate a professional attitude in addressing patients’ emotional and psychological needs related to dermatological conditions

The objectives encompass both theoretical knowledge and practical skills required to diagnose, manage, and treat skin and sexually transmitted diseases. In addition to the learning objectives, a separate list was compiled, consisting of diagnoses and medications covering a broad spectrum of inflammatory skin disorders and skin tumours as well as sexually transmitted infections.

A total of 124 diagnoses and medications were included in the final list of learning objectives and the level of knowledge was influenced both by feedback from reviewers and invited colleagues from the primary health care setting (Appendices S1, S3 [*Swedish version*] and S4). The expected level of knowledge for most entries was generally classified as either basic knowledge or composite/multifaceted knowledge. The highest level of knowledge recorded in the assessment was related knowledge, while no diagnosis or concept reached the highest category of transferable/extended knowledge. Nine conditions were classified under the “related knowledge” level: psoriasis, atopic dermatitis, acne, rosacea, perioral dermatitis, the nevus concept, melanocytic nevi, melanoma, and sexually transmitted chlamydia.

## DISCUSSION

A successful core curriculum in medical education is typically built on 4 key pillars: (a) clearly defined learning objectives that are accepted by both specialists and generalists; (b) evidence-based and context-appropriate educational methods for delivering the content; (c) adequate and valid assessments to ensure that students achieve the intended outcomes; and (d) long-term follow-up and evaluation of the curriculum to assess its effectiveness and guide future improvements. (4, 5) The present project aimed to establish the first of these components within the field of dermatology and venereology in Sweden by developing a consensus-based set of national learning objectives.

This manuscript represents a foundational step toward establishing a unified national framework for learning objectives in dermatology and venereology across medical education programmes in Sweden. Our intention is that the level and the learning objectives outlined herein should reflect the knowledge expected of all licensed physicians in Sweden. We acknowledge that this assessment is ambitious, and in this context it is important to recognize that instruction in dermatology and venereology is typically limited to approximately 2–3 weeks in total at the various universities in Sweden. The model of national learning objectives is an internationally recognized approach in medical education. Comparable national undergraduate learning objectives exist, notably the British Association of Dermatologists’ (BAD) Undergraduate Curriculum aligned with the UK Medical Licensing Assessment (6), the American Academy of Dermatology’s (AAD) Basic Dermatology Curriculum for medical students (7), and Canadian efforts led by the Canadian Professors of Dermatology (8) to define national undergraduate objectives. Moreover, recent studies in Australia, Germany, and Switzerland discuss undergraduate dermatology curricula and highlight the value of standardized national learning objectives (2, 9).

To ensure that our set of learning objectives does not become merely a theoretical exercise, it is essential in the next phase to implement a strategy that keeps the document continuously updated and visible. In this context, an important first step is to align this national consensus on learning objectives with the syllabus at each university. While the present project focuses on the declared curriculum, it is important to acknowledge that discrepancies may arise between the declared, taught, and learned curricula (10). Even when national learning objectives are clearly defined, variations in teaching methods, clinical environments, and individual educator priorities may lead to differences in what is actually taught and ultimately learned by students (11). This tension underscores the need for alignment between learning objectives, educational activities, and assessment methods. Future work should therefore include evaluation of how these objectives are implemented in practice, as well as how students perceive and internalize them, to ensure that the intended outcomes of the curriculum are truly achieved. Moreover, further pedagogical research is important to build on these findings, deepening our understanding of how the curriculum influences learning processes and long-term professional competence.

While the duration of undergraduate dermatology and venereology courses varies across universities (e.g., 9 vs 15 days in total), the achievement of learning objectives is not determined solely by course length but rather by the pedagogical design and alignment of teaching and assessment strategies. Institutions with shorter placements commonly employ structured, outcome-based curricula supplemented by preparatory e-learning modules, focused clinical exposure, and supervised case-based teaching to ensure attainment of core competencies. Longer placements, in contrast, allow for greater experiential depth through repeated patient encounters, integrated seminars, and reflective practice. It has been concluded that it is unrealistic to expect dermatology students to acquire many diagnostic skills in a short course (as in the new study programme in Sweden). Instead, teachers should ensure that students develop a framework that will enable them to analyse skin problems, to think pathophysiologically and to apply their learning in the future. Undergraduates should meet patients to receive real or simulated experiences that raise awareness of the “need to know” (12).

The use of nationally harmonized learning objectives provides a framework that supports equivalence in expected outcomes despite differences in course duration. This alignment of content, learning activities, and assessment criteria enables comparable competency development across institutions while allowing necessary flexibility in local curricular implementation.

When developing learning objectives, certain elements or procedures may inadvertently receive less emphasis than others. In a scoping review of the literature, patient safety, disease burden, societal needs, and inadequate preparedness of medical graduates were found to be drivers when developing core curricula (13). Junior doctors and general practitioners have reported feelings of inadequacy due to lack of undergraduate training in some specialties (e.g., dermatology) where related diseases and conditions are common in primary care settings (13). Therefore it is important to define minimum knowledge levels and skills to be attained in dermatology and venereology.

Based on feedback from peer reviewers and colleagues from primary healthcare, some learning objectives were expanded and nuanced to ensure a more comprehensive scope. In particular, the clarification of learning objective K1 guarantees that students gain a solid foundation in skin biology – an essential prerequisite for understanding the pathogenesis of dermatological and venereological diseases. This encompasses key concepts such as skin homeostasis, barrier function, cutaneous immunology, and vitamin D synthesis, all of which play critical roles in maintaining health and contributing to disease mechanisms. Another such element is the inclusion of surgical procedures, which currently receive minimal emphasis in the existing version of the objectives. Therefore, it is important to address this aspect in future iterations. Ideally, students should have the opportunity to practise basic surgical skills, such as simple elliptical excisions, during their dermatology and venereology studies. For each university, it must be explicitly clear where students will receive training in skills like skin suturing if these are not covered within the dermatology and venereology curriculum, acknowledging that this arrangement may vary from one institution to another.

As an important limitation, we acknowledge that employing a more rigorous Delphi consensus methodology might have produced somewhat different outcomes. It should be noted that GPs were not initially invited during the first round of feedback on the listed learning objectives. Instead, they were involved later in the process at the request of the reviewers. We did not adopt a systematic approach in selecting GPs; instead, we relied on the listed co-authors to invite relevant stakeholders. Naturally, involving a broader range of GPs might have resulted in a different result.

Moreover, we recognize that integrating comments from medical students and resident physicians in dermatology and venereology could have made the core curriculum even more robust and comprehensive. We recognize the factors above as methodological constraints and suggest that future work could adopt such an approach to further strengthen and refine the consensus process.

Nevertheless, the present document provides a valuable foundation for future research, offering a structured framework and empirically informed starting point from which subsequent studies can further explore, validate, and expand upon the proposed objectives and their implementation.

It is important that students genuinely understand the learning objectives and the process by which they have been developed. Our experience suggests that students are more likely to achieve the intended learning outcomes when these are accompanied by clear clinical examples (12). This is particularly important for abstract learning objectives. A shared understanding of how the objectives should be applied, interpreted, and emphasized during teaching is crucial. It is important that course coordinators at the universities highlight the learning objectives at the beginning of their courses. Equally important is to remain receptive to student feedback and actively seek input on how it might be further improved. Future involvement of students not only as recipients but also as co-creators of educational content and feedback mechanisms may further enhance the relevance, accessibility, and long-term acceptance of the learning objectives.

Another key aspect of this initiative is that it establishes a clearly defined, shared set of objectives, which can help foster consensus on the level of competence expected of medical students at the point of licensure. Agreement among faculty across institutions on what is expected from students also provides a vital foundation for constructive alignment (14). Importantly, the developed learning objectives can serve as a source of inspiration for the design of educational activities. An important next step is to systematically survey the teaching components offered at the various institutions, enabling mutual support and the exchange of ideas. Shared learning objectives facilitate collaboration among universities, allowing for the exchange of experiences and the recommendation of effective teaching methods that help ensure the objectives are met.

Equally important is the establishment of consistent assessment and feedback practices across institutions, which support student learning progression in the most effective way. Such collaboration can lead to a more efficient and resource-conscious education, with institutions drawing on each other’s experiences and expertise to enhance both teaching and student learning outcomes. Additionally, the exchange of proposed questions for theoretical knowledge assessments between institutions will be beneficial. In this way, robust learning objectives, broadly supported across institutions, may contribute to improved reliability and validity of examinations.

This manuscript presents the learning objectives in both English and Swedish, which constitutes a notable strength. The inclusion of an English version not only supports the internationalization of medical education but also facilitates cross-border comparison and research related to learning objectives in dermatology and venereology. Moreover, this bilingual approach aligns with the overarching goals of the new medical programme to harmonize with European standards and to clearly articulate the expected competencies.

In summary, we believe it is essential that dermatology and venereology are afforded adequate attention and resources within the new 6-year medical programme qualifying for licensure. Continued national efforts to coordinate and advance medical education in Sweden are likewise of critical importance. We hope our initiative will inspire similar efforts in other countries to develop national learning objectives for licensed physicians not only within our specialty, but also in others. In our view, the establishment of overarching national learning objectives across institutions represents a highly worthwhile goal. Moreover, the development of a Nordic consensus document would be particularly desirable, as it would enable collaboration among countries with comparable healthcare systems and educational structures. Such cross-national coordination could lay a robust foundation for harmonized and high-quality medical education throughout the Nordic region. Finally, in an era where rapid medical advances and emerging technologies like large language models (LLMs) are reshaping how clinical knowledge is accessed and applied, proactively maintaining updated, unified learning objectives through national collaboration is not only essential for ensuring educational consistency and quality – it is also a key aspiration that supports the evolving role of universities and educators in medical training.

## Supplementary Material


