# Supplementary material for: National Learning Objectives in Dermatology and Venereology for the New Swedish Medical Programme
Source: Acta Derm Venereol. 2026 Jan 23;106:44059. doi: 10.2340/actadv.v106.44059 (PMC12836398; doi:10.2340/actadv.v106.44059)
Supplement: Supplementary file 1 [file ActaDV-106-44059-s1.pdf]

Supplementary material has been published as submitted. It has not been copyedited, or typeset by Acta Dermato-Venereologica

APPENDIX S1 - Detailed list of diagnoses, medications, and concepts.

English version

**Genodermatoses:**	**Level of knowledge**
Keratosis pilaris	S1. Basic Knowledge
Ichthyosis	S1. Basic Knowledge
Epidermolysis bullosa	S1. Basic Knowledge
Neurofibromatosis type 1	S1. Basic Knowledge
Palmoplantar keratoderma	S1. Basic Knowledge
Darier's disease	S1. Basic Knowledge
Hailey-Hailey disease	S1. Basic Knowledge
Xeroderma pigmentosum	S1. Basic Knowledge
Albinism	S1. Basic Knowledge
**Inflammatory skin diseases:**	**Level of knowledge**
Psoriasis	S3. Relational Knowledge
Pustulosis palmoplantaris	S2. Multiple/Integrated Knowledge
Pityriasis rosea	S2. Multiple/Integrated Knowledge
Lichen planus	S1. Basic Knowledge
Contact dermatitis (allergic, non-allergic)	S2. Multiple/Integrated Knowledge
Atopic dermatitis	S3. Relational Knowledge
Seborrheic dermatitis	S2. Multiple/Integrated Knowledge
Stasis dermatitis	S2. Multiple/Integrated Knowledge
Lichen simplex chronicus (neurodermatitis)	S2. Multiple/Integrated Knowledge
Intertriginous eczema	S2. Multiple/Integrated Knowledge
Diaper dermatitis	S2. Multiple/Integrated Knowledge
Scarring genital dermatoses including lichen sclerosus	S1. Basic Knowledge
**Urticaria and Other Cutaneous Reaction Patterns:**	**Level of knowledge**
Acute and chronic urticaria, including angioedema	S2. Multiple/Integrated Knowledge
Erythema nodosum	S1. Basic Knowledge
**Drug eruptions:**	**Level of knowledge**
Drug exanthema	S2. Multiple/Integrated Knowledge
Erythema multiforme with or without mucosal involvement	S2. Multiple/Integrated Knowledge
Fixed drug eruption	S2. Multiple/Integrated Knowledge

**Diseases of Blood and Lymphatic Vessels:**	**Level of knowledge**
Leg ulcers (venous, arterial, and neuropathic)	S2. Multiple/Integrated Knowledge
Vasculitic diseases with cutaneous presentation	S1. Basic Knowledge
Nevus simplex	S1. Basic Knowledge
Nevus flammeus	S2. Multiple/Integrated Knowledge
Infantile hemangioma	S2. Multiple/Integrated Knowledge
Telangiectasias	S1. Basic Knowledge
Spider telangiectasia	S1. Basic Knowledge
**Connective tissue diseases, blistering dermatoses, and cutaneous signs of systemic diseases:**	**Level of knowledge**
Cutaneous lupus erythematosus	S2. Multiple/Integrated Knowledge
Morfea	S1. Basic Knowledge
Bullous pemphigoid	S2. Multiple/Integrated Knowledge
Pemphigus vulgaris	S1. Basic Knowledge
Cutaneous manifestations of lupus erythematosus	S1. Basic Knowledge
Dermatomyositis	S1. Basic Knowledge
Dermatitis herpetiformis	S1. Basic Knowledge
Pyoderma gangrenosum	S1. Basic Knowledge
Granuloma annulare	S2. Multiple/Integrated Knowledge
Xanthelasma	S1. Basic Knowledge
Cutaneous manifestations of diabetes mellitus	S2. Multiple/Integrated Knowledge
Cutaneous manifestations of liver cirrhosis	S2. Multiple/Integrated Knowledge
Cutaneous manifestations of hematological diseases	S1. Basic Knowledge
Cutaneous manifestations in organ transplant recipients	S1. Basic Knowledge
**Hair, Nails, and Adnexal Structures:**	**Level of knowledge**
Acne	S3. Relational Knowledge
Rosacea	S3. Relational Knowledge
Perioral dermatitis	S3. Relational Knowledge
Hair loss	S2. Multiple/Integrated Knowledge
Nail changes in psoriasis	S1. Basic Knowledge
Hidradenitis suppurativa	S2. Multiple/Integrated Knowledge
Hyperhidrosis	S2. Multiple/Integrated Knowledge
Hirsutism	S1. Basic Knowledge
Hypertrichosis	S1. Basic Knowledge
**Infections with Cutaneous Manifestations:**	**Level of knowledge**
Infections caused by streptococci	S2. Multiple/Integrated Knowledge
Infections caused by staphylococci	S2. Multiple/Integrated Knowledge
Infections caused by *Borrelia*	S2. Multiple/Integrated Knowledge
Infections caused by human papillomavirus	S2. Multiple/Integrated Knowledge

Infections caused by herpes simplex virus	S2. Multiple/Integrated Knowledge
Infections caused by varicella-zoster virus	S2. Multiple/Integrated Knowledge
Molluscum contagiosum	S2. Multiple/Integrated Knowledge
Infections caused by dermatophytes	S2. Multiple/Integrated Knowledge
Infections caused by yeasts	S2. Multiple/Integrated Knowledge
**Bites and Infestations:**	**Level of knowledge**
Scabies	S2. Multiple/Integrated Knowledge
Head lice, body lice, and pubic lice	S1. Basic Knowledge
Other bite reactions (body lice, fleas, mites)	S1. Basic Knowledge
**Photodermatology and Pigmentary Disorders:**	**Level of knowledge**
Skin diseases that improve or worsen with UV radiation	S1. Basic Knowledge
Hyperpigmentation and hypopigmentation	S1. Basic Knowledge
Vitiligo	S2. Multiple/Integrated Knowledge
Congenital dermal melanocytosis	S1. Basic Knowledge
**Skin Tumors:**	**Level of knowledge**
The nevus concept	S3. Relational Knowledge.
Congenital melanocytic nevi	S2. Multiple/Integrated Knowledge
Melanocytic nevi	S3. Relational Knowledge
Nevus sebaceus	S1. Basic Knowledge
Seborrheic keratosis / solar lentigo	S2. Multiple/Integrated Knowledge
Pilomatricoma	S1. Basic Knowledge
Angiomas/hemangiomas	S1. Basic Knowledge
Pyogenic granuloma (lobular capillary hemangioma)	S2. Multiple/Integrated Knowledge
Dermatofibroma	S2. Multiple/Integrated Knowledge
Keloids	S1. Basic Knowledge
Basal cell carcinoma	S2. Multiple/Integrated Knowledge
Squamous cell carcinoma (including keratoacanthoma)	S2. Multiple/Integrated Knowledge
Squamous cell carcinoma *in situ*	S2. Multiple/Integrated Knowledge
Actinic keratosis	S2. Multiple/Integrated Knowledge
Melanoma	S3. Relational Knowledge
Cutaneous lymphomas	S1. Basic Knowledge
Cutaneous metastases	S1. Basic Knowledge
**Acute Dermatologic Conditions:**	**Level of knowledge**
Staphylococcal scalded skin syndrome (SSSS)	S1. Basic Knowledge
Toxic epidermal necrolysis (TEN) – Stevens-Johnson syndrome (SJS)	S2. Multiple/Integrated Knowledge
Generalized pustular psoriasis (GPP)	S1. Basic Knowledge
Eczema herpeticum	S1. Basic Knowledge
Erythroderma	S1. Basic Knowledge

**Venereology:**	**Level of knowledge**
Chlamydia, including lymphogranuloma venereum	S3. Relational Knowledge.
Gonorrhoea	S2. Multiple/Integrated Knowledge
Mycoplasma genitalium	S2. Multiple/Integrated Knowledge
Non-specific urethritis/cervicitis	S2. Multiple/Integrated Knowledge
Genital human papillomavirus (HPV) infection	S2. Multiple/Integrated Knowledge
Genital herpes	S2. Multiple/Integrated Knowledge
Syphilis	S2. Multiple/Integrated Knowledge
Trichomonas vaginalis	S1. Basic Knowledge
Vulvovaginal fungal infections	S2. Multiple/Integrated Knowledge
Other causes of genital ulcers than herpes simplex and syphilis	S1. Basic Knowledge
Cutaneous manifestations of HIV/AIDS	S1. Basic Knowledge
Mpox	S1. Basic Knowledge
**Systemic Medications:**	**Level of knowledge**
Acitretin	S1. Basic Knowledge
Methotrexate	S1. Basic Knowledge
Cyclosporine	S1. Basic Knowledge
Systemic glucocorticoids (i.e., Prednisolone)	S2. Multiple/Integrated Knowledge
Tetracycline	S2. Multiple/Integrated Knowledge
Dapsone	S1. Basic Knowledge
Isotretinoin	S1. Basic Knowledge
Antihistamines	S2. Multiple/Integrated Knowledge
Biologic agents for psoriasis / atopic dermatitis	S1. Basic Knowledge
Biologic agents for chronic urticaria	S1. Basic Knowledge
HIV pre-exposure prophylaxis (PrEP)	S1. Basic Knowledge
JAK-inhibitors	S1. Basic Knowledge
**Topical and Injected Medications:**	**Level of knowledge**
Emollients	S2. Multiple/Integrated Knowledge
Topical corticosteroids	S2. Multiple/Integrated Knowledge
Calcineurin inhibitors	S2. Multiple/Integrated Knowledge
Antifungal medications	S2. Multiple/Integrated Knowledge
Retinoids	S2. Multiple/Integrated Knowledge
Botulinum toxin	S1. Basic Knowledge

APPENDIX S2 - Lärandemål för dermatologi och venereologi för det nya läkarprogrammet

Swedish version

Kunskap och förståelse
**K1.** Kunskap om etiologi och patogenes vid olika hud- och könssjukdomars (*se bilaga 1 för vägledning)*, inklusive grundläggande principer för hudbiologi (dvs. struktur och funktion) såsom hudens homeostas, hudbarriärens funktion, kutan immunologi och syntes av vitamin D.<br>**K2.** Förmåga att kliniskt känna igen och särskilja olika inflammatoriska hudsjukdomar, hudtumörer och könssjukdomar samt deras karaktäristika för att ställa korrekt diagnos (*se bilaga 1 för vägledning)*.<br>**K3.** Förståelse för farmakologiska egenskaper samt biverkningsprofil för de vanligaste läkemedel som används för att behandla hud- och könssjukdomar (*se bilaga 1 för vägledning)*.<br>**K4.** Kunskap om olika diagnostiska metoder och deras användning för att bedöma hud- och könssjukdomar.<br>**K5.** Förståelse för hur genetiska och miljömässiga faktorer påverkar uppkomsten och förloppet av hud- och könssjukdomar.<br>**K6.** Grundläggande kunskap om epidemiologi och förekomst av olika hud- och könssjukdomar både nationellt och globalt.<br>**K7.** Förståelse för evidensbaserade riktlinjer och deras roll vid utvärdering och behandling av hud- och könssjukdomar.<br>**K8.** Förståelse för hur hud- och könssjukdomar manifesterar sig olika hos patienter med olika hudtyper, åldrar och kön, och hur detta påverkar diagnostik och behandling.<br>**K9.** Kunskap om kulturella och sociala faktorers påverkan på patienters upplevelser av hud- och könssjukdomar.<br>**K10.** Förståelse för multidisciplinära tillvägagångssätt och samarbete mellan olika medicinska specialiteter för att behandla komplexa hud- och könssjukdomar hos olika patientgrupper.<br>**K11.** Kunskap om akuta dermatologiska tillstånd (*se bilaga 1 för vägledning)*.<br>**K12.** Kunskap om förebyggande åtgärder och patientutbildning för att minimera hud- och könssjukdomars risker och följder.<br>**K13.** Förståelse för hur hud- och könssjukdomar kan påverka patienters hälsorelaterade livskvalitet.<br>**K14.** Kunskap om etiska och juridiska aspekter inom hud- och könssjukvård, inklusive smittskyddslagstiftning, patientsekretess och samtycke till behandling.<br>**K15.** Förståelse för remissprocessen, inklusive de nödvändiga detaljerna och formatet för att underlätta en god kommunikation mellan primärvård och specialistsjukvård.<br>**K16.** Kunskap om korrekt användning av terminologi inom hud- och könssjukvård för att tydligt och enhetligt kommunicera med kollegor och patienter samt underlätta effektivt informationsutbyte och dokumentation.<br>**K17.** Förståelse om principerna gällande ljusbehandlingar (smalbands-UVB, UVA) och ljusbaserade metoder (LASER, IPL) som används inom hud- och könssjukvård.<br>**K18.** Förståelse för arbetet med smittspårning och hur den utförs.<br>**K19.** Kunskap om olika terapeutiska procedurer, inklusive kirurgiska ingrepp och frysbehandling, för olika hud- och könssjukdomar.

Färdighet och förmåga
**F1.** Färdighet att genomföra och dokumentera en hudundersökning för att identifiera olika hudlesioner inklusive inflammatoriska hudsjukdomar, hudtumörer samt könssjukdomar.<br>**F2.** Kunna utföra en stansbiopsi i huden.<br>**F3.** Färdighet att använda olika diagnostiska verktyg och metoder, såsom dermatoskopi och ankel/arm-index.<br>**F4.** Förmåga att formulera relevanta differentialdiagnoser baserat på kliniska observationer och laboratorieresultat.<br>**F5.** Färdighet att hantera och följa upp patienter med kroniska hud- och könssjukdomar för att övervaka sjukdomsförlopp och behandlingseffekt.<br>**F6.** Förmåga att kommunicera personcentrerat med patienter om deras hud- och könssjukdomar, behandlingsalternativ och prognos.<br>**F7.** Färdighet att utföra en effektiv och korrekt bedömning av hud- och könssjukdomar hos patienter med olika hudfärg.<br>**F8.** Färdighet att hantera och behandla akuta dermatologiska tillstånd (*se bilaga 1 för vägledning)*.<br>**F9.** Förmåga att utbilda och ge råd till patienter om förebyggande åtgärder för att minska risken för hud- och könssjukdomar.<br>**F10**. Förmåga att bedöma och handlägga smittsamma hud- och könssjukdomar för att förhindra spridning och minimera riskerna för patienter och samhället.<br>**F11.** Färdighet att anpassa behandlingsstrategier och terapier utifrån individuella patienters behov och bakomliggande faktorer (kloka kliniska val).<br>**F12.** Färdighet att effektivt bedöma och dokumentera hud- och könssjukdomar för att skriva välgrundade och precisa remisser till specialistsjukvård.
Värderingsförmåga och förhållningssätt
**V1.** Identifiera och reflektera över betydelsen av patientcentrerad vård för att förbättra behandlingsresultat inom hud- och könssjukvård.<br>**V2.** Visa ett reflekterat, professionellt förhållningssätt gentemot patientens kulturella och sociala bakgrund vid diagnostik och behandling av hudrelaterade tillstånd.<br>**V3.** Uppvisa ett professionellt förhållningssätt gentemot etiska frågor och integritetsfrågor som kan uppstå vid behandling och hantering av patientinformation inom hud- och könssjukvård.<br>**V4.** Identifiera och reflektera över hur socioekonomiska faktorer kan påverka tillgången till vård och behandling inom hud- och könssjukvård.<br>**V5.** Uppvisa ett professionellt förhållningssätt i hanteringen av patienters emotionella och psykologiska behov i samband med hudrelaterade tillstånd.

APPENDIX S3 - Detaljerad lista med diagnoser, läkemedel och begrepp.

Swedish version

**Gendermatoser:**	**Nivå av kunskap**
Keratosis pilaris	S1. Enkla kunskaper
Iktyos	S1. Enkla kunskaper
Epidermolysis bullosa	S1. Enkla kunskaper
Neurofibromatos typ 1	S1. Enkla kunskaper
Palmoplantar keratodermi	S1. Enkla kunskaper
Dariers sjukdom	S1. Enkla kunskaper
Hailey-Hailey	S1. Enkla kunskaper
Xeroderma pigmentosum	S1. Enkla kunskaper
Albinism	S1. Enkla kunskaper
**Inflammatoriska hudsjukdomar:**	**Nivå av kunskap**
Psoriasis	S3. Relaterad kunskap
Pustulosis palmoplantaris	S2. Flerfaldiga/sammansatta kunskaper
Pityriasis rosea	S2. Flerfaldiga/sammansatta kunskaper
Lichen planus	S1. Enkla kunskaper
Kontakteksem (allergiskt, icke-allergiskt)	S2. Flerfaldiga/sammansatta kunskaper
Atopisk dermatit	S3. Relaterad kunskap
Seborroiskt eksem	S2. Flerfaldiga/sammansatta kunskaper
Staseksem	S2. Flerfaldiga/sammansatta kunskaper
Lichen simplex chronicus (neurodermatit)	S2. Flerfaldiga/sammansatta kunskaper
Intertriginöst eksem	S2. Flerfaldiga/sammansatta kunskaper
Blöjdermatit	S2. Flerfaldiga/sammansatta kunskaper
Ärrbildande genitala dermatoser inklusive lichen sclerosus	S1. Enkla kunskaper
**Urtikaria och andra reaktionsmönster i huden:**	**Nivå av kunskap**
Akut och kronisk urtikaria samt angioödem	S2. Flerfaldiga/sammansatta kunskaper
Erythema nodosum	S1. Enkla kunskaper
**Läkemedelsutslag:**	**Nivå av kunskap**
Läkemedelsexantem	S2. Flerfaldiga/sammansatta kunskaper
Erythema multiforme med eller utan slemhinneengagemang	S2. Flerfaldiga/sammansatta kunskaper
Fixt läkemedelsutslag	S2. Flerfaldiga/sammansatta kunskaper
**Sjukdomar i blod- och lymfkärl:**	**Nivå av kunskap**
Bensår (venösa, arteriella och neuropatiska)	S2. Flerfaldiga/sammansatta kunskaper
Vaskulitsjukdomar med presentation i huden	S1. Enkla kunskaper
Nevus simplex	S1. Enkla kunskaper

Nevus flammeus	S2. Flerfaldiga/sammansatta kunskaper
Infantilt hemangiom	S2. Flerfaldiga/sammansatta kunskaper
Telangiektasier	S1. Enkla kunskaper
Spider telangiektasi	S1. Enkla kunskaper
**Bindvävssjukdomar, blåsdermatoser samt hudsymtom vid systemsjukdomar:**	**Nivå av kunskap**
Kutan lupus erythematosus	S2. Flerfaldiga/sammansatta kunskaper
Morfea	S1. Enkla kunskaper
Bullös pemfigoid	S2. Flerfaldiga/sammansatta kunskaper
Pemfigus vulgaris	S1. Enkla kunskaper
Kutana manifestationer vid lupus erythematosus	S1. Enkla kunskaper
Dermatomyosit	S1. Enkla kunskaper
Dermatitis herpetiformis	S1. Enkla kunskaper
Pyoderma gangrenosum	S1. Enkla kunskaper
Granuloma annulare	S2. Flerfaldiga/sammansatta kunskaper
Xantelasma	S1. Enkla kunskaper
Hudmanifestationer vid diabetes mellitus	S2. Flerfaldiga/sammansatta kunskaper
Hudmanifestationer vid levercirrhos	S2. Flerfaldiga/sammansatta kunskaper
Hudmanifestationer vid hematologiska sjukdomar	S1. Enkla kunskaper
Hudmanifestationer hos organtransplanterade	S1. Enkla kunskaper
**Hår, naglar och adnexorgan:**	**Nivå av kunskap**
Akne	S3. Relaterad kunskap
Rosacea	S3. Relaterad kunskap
Perioral dermatit	S3. Relaterad kunskap
Håravfall	S2. Flerfaldiga/sammansatta kunskaper
Nagelförändringar vid psoriasis	S1. Enkla kunskaper
Hidradenitis suppurativa	S2. Flerfaldiga/sammansatta kunskaper
Hyperhidros	S2. Flerfaldiga/sammansatta kunskaper
Hirsutism	S1. Enkla kunskaper
Hypertrichos	S1. Enkla kunskaper
**Infektioner med hudsymtom:**	**Nivå av kunskap**
Infektioner orsakade av streptokocker	S2. Flerfaldiga/sammansatta kunskaper
Infektioner orsakade av stafylokocker	S2. Flerfaldiga/sammansatta kunskaper
Infektioner orsakade av borrelia	S2. Flerfaldiga/sammansatta kunskaper
Infektioner orsakade av humant papillomvirus	S2. Flerfaldiga/sammansatta kunskaper
Infektioner orsakade av herpes simplexvirus	S2. Flerfaldiga/sammansatta kunskaper
Infektioner orsakade av varicella-zostervirus	S2. Flerfaldiga/sammansatta kunskaper
Mollusker	S2. Flerfaldiga/sammansatta kunskaper
Infektioner orsakade av dermatofyter	S2. Flerfaldiga/sammansatta kunskaper

Infektioner orsakade av jästsvampar	S2. Flerfaldiga/sammansatta kunskaper
**Bett och infestationer:**	**Nivå av kunskap**
Skabb	S2. Flerfaldiga/sammansatta kunskaper
Huvud-, vägg- och flatlöss	S1. Enkla kunskaper
Andra bettreaktioner (klädlöss, loppor, kvalster)	S1. Enkla kunskaper
**Fotodermatologi och pigmentförändringar:**	**Nivå av kunskap**
Hudsjukdomar som förbättras/försämras av UV-strålning	S1. Enkla kunskaper
Hyper- och hypopigmenteringar	S1. Enkla kunskaper
Vitiligo	S2. Flerfaldiga/sammansatta kunskaper
Kongenital dermal melanos	S1. Enkla kunskaper
**Hudtumörer:**	**Nivå av kunskap**
Nevusbegreppet	S3. Relaterad kunskap.
Medfödda melanocytära nevi	S2. Flerfaldiga/sammansatta kunskaper
Melanocytära nevi	S3. Relaterad kunskap
Nevus sebaceus	S1. Enkla kunskaper
Seborroisk keratos/lentigo solaris	S2. Flerfaldiga/sammansatta kunskaper
Pilomatrixom	S1. Enkla kunskaper
Angiom/hemangiom	S1. Enkla kunskaper
Pyogent granulom (lobulärt kapillärt hemangiom)	S2. Flerfaldiga/sammansatta kunskaper
Dermatofibrom	S2. Flerfaldiga/sammansatta kunskaper
Keloider	S1. Enkla kunskaper
Basalcellscancer	S2. Flerfaldiga/sammansatta kunskaper
Skivepitelcancer (inklusive keratoakantom)	S2. Flerfaldiga/sammansatta kunskaper
Skivepitelcancer *in situ*	S2. Flerfaldiga/sammansatta kunskaper
Aktinisk keratos	S2. Flerfaldiga/sammansatta kunskaper
Melanom	S3. Relaterad kunskap
Kutana lymfom	S1. Enkla kunskaper
Kutana metastaser	S1. Enkla kunskaper
Akuta dermatologiska tillstånd	**Nivå av kunskap**
Staphylococcal scalded skin syndrome (SSSS)	S1. Enkla kunskaper
Toxisk epidermal nekrolys (TEN) - Stevens-Johnson syndrom (SJS)	S2. Flerfaldiga/sammansatta kunskaper
Generaliserad pustulär psoriasis (GPP)	S1. Enkla kunskaper
Eczema herpeticum	S1. Enkla kunskaper
Erythrodermi	S1. Enkla kunskaper
Venereologi	**Nivå av kunskap**
Klamydia inklusive lymfogranuloma venereum	S3. Relaterad kunskap.

Gonorré	S2. Flerfaldiga/sammansatta kunskaper
Mycoplasma genitalium	S2. Flerfaldiga/sammansatta kunskaper
Ospecifik uretrit/cervicit	S2. Flerfaldiga/sammansatta kunskaper
Genital papillomvirusinfektion	S2. Flerfaldiga/sammansatta kunskaper
Genital herpes	S2. Flerfaldiga/sammansatta kunskaper
Syfilis	S2. Flerfaldiga/sammansatta kunskaper
Trichomonas vaginalis	S1. Enkla kunskaper
Vulvovaginala svampinfektioner	S2. Flerfaldiga/sammansatta kunskaper
Andra orsaker till genitala sår än herpes simplex och syfilis	S1. Enkla kunskaper
Hudsymtom vid HIV/AIDS	S1. Enkla kunskaper
Mpox	S1. Enkla kunskaper
Systemiska läkemedel	**Nivå av kunskap**
Acitretin	S1. Enkla kunskaper
Methotrexate	S1. Enkla kunskaper
Cyclosporin	S1. Enkla kunskaper
Systemiska glykokortikoider (i.e. Prednisolon)	S2. Flerfaldiga/sammansatta kunskaper
Tetracyklin	S2. Flerfaldiga/sammansatta kunskaper
Dapson	S1. Enkla kunskaper
Isotretinoin	S1. Enkla kunskaper
Antihistaminer	S2. Flerfaldiga/sammansatta kunskaper
Biologiska läkemedel mot psoriasis / atopisk dermatit	S1. Enkla kunskaper
Biologiska läkemedel mot kronisk urtikaria	S1. Enkla kunskaper
Preexpositionsprofylax för HIV	S1. Enkla kunskaper
JAK-hämmare	S1. Enkla kunskaper
Topikala och injicerade läkemedel	**Nivå av kunskap**
Mjukgörande	S2. Flerfaldiga/sammansatta kunskaper
Topikala kortisonpreparat	S2. Flerfaldiga/sammansatta kunskaper
Calcineurinhämmare	S2. Flerfaldiga/sammansatta kunskaper
Antimykotiska läkemedel	S2. Flerfaldiga/sammansatta kunskaper
Retinoider	S2. Flerfaldiga/sammansatta kunskaper
Botulinumtoxin	S1. Enkla kunskaper

APPENDIX S4 – Tabulated constructive feedback for all invited physicians working in primary health care.

Primary healthcare physician 1

- Finds the learning objectives comprehensive and well balanced between common and specialist-level conditions.
- Suggests adding understanding of treatment adherence—factors influencing it and its impact on disease outcomes.
- Emphasizes comorbidity, noting how other diseases can predispose to or influence skin disease progression.

Primary healthcare physician 2

- Praises the initiative to develop a consensus document.
- Suggests using the term "person-centered communication" instead of "empathetic communication."
- Recommends elevating contact dermatitis and seborrheic eczema to the same level (*i.e.,* S3) as atopic dermatitis.
- Questions the high level for lichen planus and suggests adjusting it.
- Suggests perioral dermatitis (POD) should be grouped with acne and rosacea.
- Vitiligo could be lower (S1).
- Recommends better knowledge of tetracyclines and antihistamines, as these are often prescribed in primary care.

Primary healthcare physician 3

- Describes the objectives as rigorous and well developed.
- Suggests clarifying the grading definitions for diagnostic, prognostic, and therapeutic knowledge.
- Recommends clarifying differences between "knowledge" and "understanding" in Appendix S1.
- Suggests potential merging or simplification of overlapping learning objectives.
- Questions the expected precision for referral writing at undergraduate level—might be too advanced.
- Wonders why transplant-related skin manifestations have higher level than more common systemic diseases (e.g. diabetes, cirrhosis).

Primary healthcare physician 4

- Finds the objectives appropriate for general practitioners but perhaps too extensive for all physicians.
- Warns that excessive detail may obscure key messages.
- Suggests lowering one level the required knowledge for inflammatory skin diseases and skin/adnexal/organ-related conditions.

Primary healthcare physician 5

- Believes knowledge requirements are too high for rare diseases such as epidermolysis bullosa, Darier disease, Hailey-Hailey disease, and morphea, and suggests removing these.
- Recommends lowering to S1 for nevus flammeus, infantile hemangioma, Stevens-Johnson syndrome, and skin manifestations in organ transplant recipients.
- Suggests raising to S2 for xanthelasma.

Primary healthcare physician 6

- Missing objectives about interprofessional collaboration and understanding roles within the clinical team.
- Should include skills in teamwork and communication with relatives as well as patients.
- Diagnosis list is appreciated but some conditions are unfamiliar and may be too long.

Primary healthcare physician 7

- Strongly positive overall; describes the work as impressive and important.
- Suggests expanding K12 to include knowledge of risks, consequences, and prevention of infertility and cervical cancer in venereology.
- Proposes raising knowledge level for seborrheic eczema, stasis dermatitis, urticaria/angioedema (S3), staphylococcal, streptococcal, and HPV infections (S3), hair loss (S3), dermatitis herpetiformis (S4), and lice infestations (higher level).
- Proposes lowering nevus flammeus and infantile hemangioma to S1.

Primary healthcare physician 8

- Praises the curriculum as comprehensive and well structured.
- Recommends more emphasis on venereology due to rising STI prevalence.
- Suggests grouping rare genodermatoses as 'rare inherited skin diseases' rather than treating each in detail.
- Notes unequal course duration across universities, which may cause competence differences.
- Describes the curriculum as ambitious, balanced, and well designed, but warns of mismatch between breadth and limited teaching time.

Primary healthcare physician 9

- Overall very positive; confirms objectives are clear, active, and assessable.
- Notes that venous ulcers should be S3, as they are common and resource-intensive in primary care.
- Suggests reconsidering prioritization of systemic drugs—some are irrelevant to primary care (e.g., biologics, acitretin, dapson).
- Recommends raising attention to lichen sclerosus—important as differential diagnosis in balanitis or phimosis.
- Notes time constraints—9 days may be too short for students to reach the stated level.
- Suggests adding learning outcomes about team collaboration with nurses and assistants and basic knowledge of wound care materials.
